# Dip-coating decoration of Ag_2_O nanoparticles on SnO_2_ nanowires for high-performance H_2_S gas sensors

**DOI:** 10.1039/d0ra02266g

**Published:** 2020-05-06

**Authors:** Tran Thi Ngoc Hoa, Nguyen Van Duy, Chu Manh Hung, Nguyen Van Hieu, Ho Huu Hau, Nguyen Duc Hoa

**Affiliations:** International Training Institute for Materials Science (ITIMS), Hanoi University of Science and Technology (HUST) No 1 - Dai Co Viet Str. Hanoi Vietnam ndhoa@itims.edu.vn hoa.nguyenduc@hust.edu.vn nguyenvanduy@itims.edu.vn; Faculty of Electrical and Electronics Engineering, Phenikaa Institute for Advanced Study, Phenikaa University Yen Nghia, Ha-Dong District Hanoi 10000 Vietnam

## Abstract

SnO_2_ nanowires (NWs) are used in gas sensors, but their response to highly toxic gas H_2_S is low. Thus, their performance toward the effective detection of low-level H_2_S in air should be improved for environmental-pollution control and monitoring. Herein, Ag_2_O nanoparticle decorated SnO_2_ NWs were prepared by a simple on-chip growth and subsequent dip-coating method. The amount of decorated Ag_2_O nanoparticles on the surface of SnO_2_ NWs was modified by changing the concentration of AgNO_3_ solution and/or dipping times. Gas-sensing measurements were conducted at various working temperatures (200–400 °C) toward different H_2_S concentrations ranging within 0.1–1 ppm. The selectivity of Ag_2_O-decorated SnO_2_ NW sensors for ammonia and hydrogen gases was tested. Results confirmed that the Ag_2_O-decorated SnO_2_ NW sensors had excellent response, selectivity, and reproducibility. The gas-sensing mechanism was interpreted under the light of energy-band bending by sulfurization, which converted the p–n junction into n–n, thereby significantly enhancing the sensing performance.

## Abstract 2

Ag_2_O nanoparticles decorated on the surface of on-chip growth SnO_2_ nanowires by a dip-coating method possessed excellent sensing performance for H_2_S gas.

## Graphical abstract

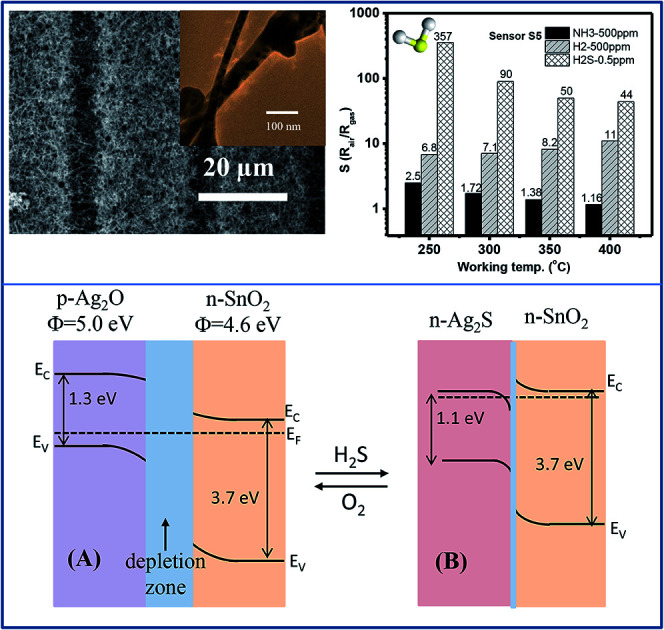

## 1. Introduction

Air pollution caused by H_2_S gas is extremely dangerous even at low concentrations (sub-ppm level) because this gas is colorless, flammable, and highly toxic.^1^ The sources of H_2_S are very diverse^2^ because it can be produced naturally from crude petroleum, oil drilling, and volcano eruption or from the bacterial decomposition of organic matter in anaerobic environments.^3^ H_2_S is also produced as a by-product in biogas plants during waste treatment.^4^ The effects of H_2_S on the human body are summarized in Table 1.^5^ The threshold odor concentration of H_2_S is about 10 ppb, but its toxic concentration range is very broad (*i.e.*, from ppb to ppm). The threshold limit of H_2_S is reportedly 0.003 ppm for 8 h of exposure.^5^ However, the permissible concentration of H_2_S recommended by the Scientific Advisory Board on Toxic Air Pollutants (USA) ranges within 20–100 ppb.^6^ Thus, effective gas sensors for detecting low levels of H_2_S under field conditions are urgent to develop.^2^

**Table 1:** Effects of exposure to H_2_S.^5^

Concentration (ppm)	Effects
0.003–0.02	Approximate threshold for odor
3–10	Obvious offensive odor
50–100	Serious eye irritation and respiratory tract irritation
100–200	Loss of smell
250–500	Fluid buildup in lungs and imminent threat to life
500	Anxiety, headache, dizziness, excessively rapid respiration, amnesia, and unconsciousness
500–1000	Immediate collapse, irregular heartbeat, neural paralysis, and respiratory paralysis leading to death

Many techniques for H_2_S detection have been developed, but metal oxide-based resistive-type gas sensors are advantageous because of their low cost, high sensitivity, real-time detection, portability, and low power consumption.^7–10^ SnO_2_ (ref. 11) is one of the most popular materials for such sensors because of its relatively high sensitivity to various gases, as well as its feasibility in functionalization to improve sensing performance.^12,13^ However, SnO_2_ has the main drawback of low response to low concentration of H_2_S^14^ and poor selectivity over air-polluting gases such as NH_3_, H_2_S, and CO.^15,16^ This problem can be solved by using heterojunctions between two dissimilar semiconducting materials, which utilizes the unique effects and leads to enhanced sensor performance.^15^ Nano-heterostructures are often utilized owing to their small size and high surface-to-volume ratio,^17,18^ and many efforts have been devoted to the fabrication of p–n heterojunctions for increasing H_2_S-sensing performance.^19–24^ The most common p–type metal oxides used to form heterojunctions with n-type SnO_2_ semiconductor are CuO,^18,25^ NiO,^26,27^ and Co_3_O_4_ (ref. 24) because of their easy sulfidation into CuS, NiS, and CoS, respectively. However, sensors with these oxides can detect H_2_S gas only at high concentrations of >10 ppm^28^ because the sulfidation of transition-metal oxides requires a high supply of sulfur source.^29^ Meanwhile, Ag_2_O has unique characteristics that enable it to functionalize SnO_2_ nanomaterials to enhance gas-sensing performance to different gases such as H_2_,^30^ ethanol,^31^ and CO.^32^ The decoration of p-type Ag_2_O on the surface of n-type SnO_2_ is advantage over the use of metallic Ag because it forms the p–n heterojunction, thus enhances the gas sensing performance.^33–35^ Ag_2_O is also reported easily converted into Ag_2_S in the presence of H_2_S^36^ because of its low free Gibbs energy for the reaction. The free Gibbs energy for conversion of Ag_2_O, CuO, and NiO into Ag_2_S, CuS, and NiS in the present of H_2_S gas is −224.7, −119.1, and −62.5 kJ mol^−1^, respectively. Therefore, decoration of Ag_2_O nanoparticles on the surface of SnO_2_ is expected to show better sensing performance such as low detection limit of H_2_S with higher sensitivity than others. However, few studies have focused on improving of H_2_S-sensing properties using Ag_2_O/SnO_2_ thin film.^33–35^ It is hard to find the related work reported on the decoration of Ag_2_O on the surface of SnO_2_ NWs for enhanced H_2_S gas despite the significantly higher stability of NWs than their thin-film counterparts.^37^ Doped thick films have shown good sensitivity to low concentrations of H_2_S but are not feasible to miniaturize.^33^ Decorated thin films present poor response to high concentrations of H_2_S.^34^ A previous work^35^ has reported extremely low response (99%) to the high H_2_S concentration of 50 ppm at 74 °C. Our group has recently reported the H_2_S-sensing characteristics of self-heated Ag-coated SnO_2_ NWs, where the decoration of Ag is realized by sputtering method.^38^ However, this method requires vacuum conditions and expensive equipment for Ag decoration, and the content of Ag_2_O nanoparticles on the surface of SnO_2_ NWs are difficult to control. Thus, a low-cost, suitable, and effective method for functionalizing p-type Ag_2_O nanoparticles with low activation energy for reversible sulfidation and oxidation, as well as enhanced H_2_S-sensing performance of SnO_2_ NWs, must be developed.

Herein, we reported the dip-coating decoration of Ag_2_O nanoparticles on the surface of on-chip-grown SnO_2_ NWs to enhance their H_2_S gas-sensing performance. Decoration was realized by dipping the sensor in AgNO_3_ solution, followed by oxidation to form Ag_2_O nanocrystals on the surface of SnO_2_ NWs. The effects of Ag_2_O content on the H_2_S gas-sensing performance of the SnO_2_ NWs were studied to maximize sensor response to H_2_S. Results demonstrated that the sensors processed excellent performance for monitoring extremely low H_2_S concentrations. The H_2_S gas-sensing mechanism of the SnO_2_ NWs functionalized with Ag_2_O nanoparticles was also discussed through the perspective of band-structure and sulfurization process.

## 2. Experimental

**Scheme 1:**
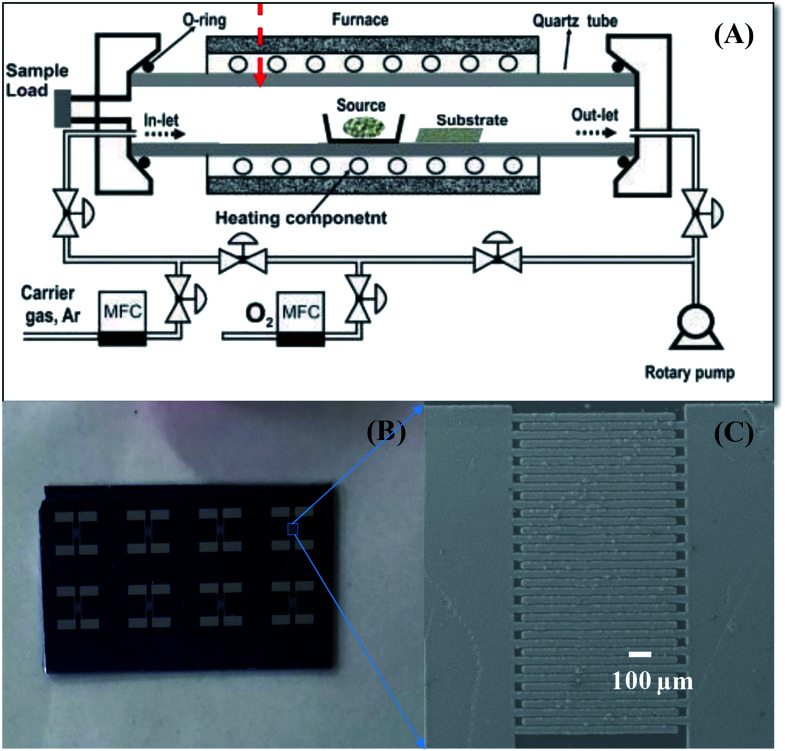
. Sensor fabrication process: (A) CVD system used to grow SnO_2_ NWs, (B) photo of sensor chips; (C) SnO_2_ NW sensor after fabrication.

The preparation of SnO_2_ NWs-based sensors has been described in our previous publication.^14^ The NW sensors were directly grown on thermally oxidized silicon substrate using a chemical vapor deposition system, as shown in Scheme 1(A).^39^ In a typical procedure, SnO_2_ NWs were grown on seeded Pt electrodes at 750 °C from a starting material of Sn powder through thermal evaporation. Growth proceeded at 750 °C for 20 min with an oxygen gas flow of 0.5 sccm and pressure of 1.8 × 10^−1^ torr. For one batch of fabrication, up to 8 sensors were obtained, as shown in Scheme 1(B). The SnO_2_ NWs were homogenously grown on the Pt electrode fingers, as shown in Scheme 1(C). The bare SnO_2_ NWs sensors were decorated with Ag_2_O nanoparticles by dip coating in AgNO_3_ solutions and subsequent annealing at 500 °C for 3 h in air. This decoration method had the advantage over the sputtering method of not requiring vacuum conditions.^38^ The density of Ag_2_O nanoparticles decorated on the surface of SnO_2_ NWs was controlled by varying the concentration of AgNO_3_ solution (0.05, 0.2, and 1 mM) and the dipping times (1, 5, and 20 times). The samples were denoted as S0, S1, S2, S3, S4, and S5 (Table 2). The morphology, chemical composition and structural characteristics of pristine and Ag_2_O-decorated SnO_2_ NWs were investigated by scanning electron microscopy (SEM; JEOL 7600F), energy-dispersive X-ray spectroscopy (EDS), high-resolution transmission electron microscopy (HRTEM; JEOL 2100F), and X-ray diffraction (XRD; D8 Advance).^3^

**Table 2:** Samples at different AgNO_3_ concentrations and dipping times

Sample	AgNO_3_ concentration (mM)	Dipping times
S0	0	0
S1	0.05	1
S2	0.2	1
S3	1	1
S4	1	5
S5	1	20

Gas-sensing properties were measured using a SourceMeter® Keithley 2602B. Details about the gas-sensing measurement system are described elsewhere.^40^ Dry air was used as reference and diluting gas. Sensor response to different H_2_S concentrations (0.1–1 ppm) at various working temperatures (200, 250, 300, 350, and 400 °C) were investigated. The selectivity among reducing gases (including ammonia and hydrogen) and the reproducibility of the sensors were also tested. During gas-sensing measurements, sensor resistance was continuously recorded, and the target gas and dry air were alternatively switched on/off. Gas response was defined as *S* = *R*_a_/*R*_g_ for the reducing gas H_2_S, where *R*_a_ and *R*_g_ are the sensor resistances in air and in target gas, respectively.^3^

## 3. Results and discussion

### 3.1. Material characterization

**Fig. 1:**
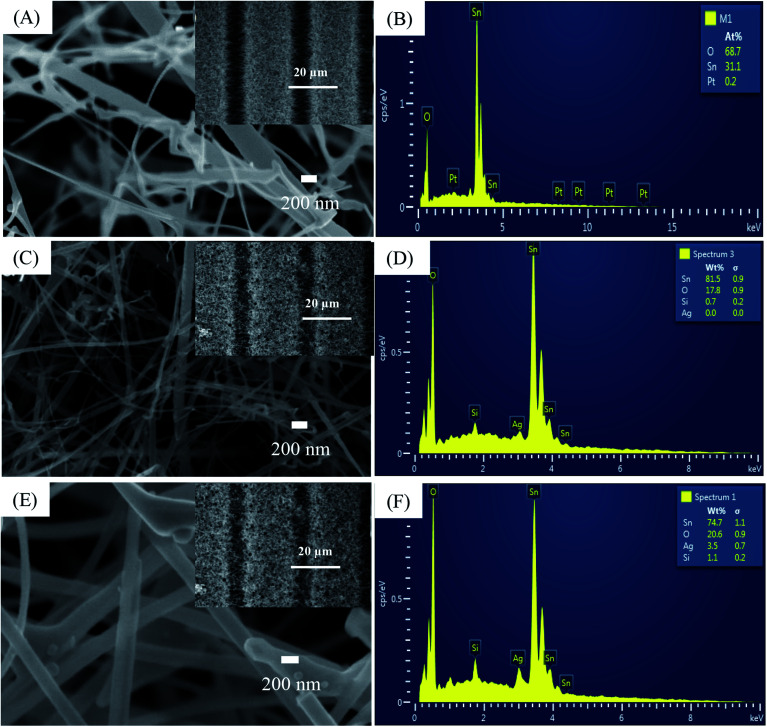
SEM images and EDS analysis of: (A and B) sensor S1 (0.05 mM); (C and D) sensor S2 (0.2 mM), (E and F) sensor S5 (1 mM). Insets are correspondent sensors' fringes.

We did not characterize all samples and instead selected sensors S1, S2, and S5 for SEM, EDS, and TEM analysis. Fig. 1(A) illustrates a SEM image of SnO_2_ NWs (S1) grown on patterned Pt electrodes. Notably, the electrode finger was 20 μm wide [inset of Fig. 1(A)]. Although the gap between two electrode fingers was 20 μm, the grown SnO_2_ NWs can still efficiently cover the gaps, as shown in the inset of Fig. 1(A). SnO_2_ NWs grew primarily on the surface of Pt electrode fingers, but their lengths were controlled sufficiently to connect between the fingers and thus act as conducting channels in the gas-sensing measurement. The average diameter of SnO_2_ NWs was approximately 70 nm. The surface of pristine SnO_2_ NWs was as smooth as that of the single crystal. This result was consistent with the growth of SnO_2_ NWs by vapor–liquid–solid mechanism.^41^ Herein, we did not use Au as catalyst during the growth of SnO_2_ NWs, so belt-like NWs were obtained at the initial state. A SnO_2_ NW comprises a single crystal, as reported in our previous article.^14^ Composition analysis of the SnO_2_ NW by EDS [Fig. 1(B)] revealed the existance of O, Sn, and Pt elements. Pt was originally from the electrode, whereas O and Sn were from the SnO_2_ NWs.

The SEM image of SnO_2_ NWs after decoration with Ag_2_O nanoparticles (S2) is presented in Fig. 1(C), whose inset is a low-magnification SEM image. The electrode fingers were covered by the SnO_2_ NWs. Ag_2_O decoration by dip coating maintained the morphology of the SnO_2_ NWs, but their surface was not as smooth as that of the pristine sample and tiny particles can be seen in the SEM images. The high-magnification SEM image revealed the presence of Ag_2_O nanoparticles on the surface of SnO_2_ NWs. EDS composition analysis of S2 [Fig. 1(D)] confirmed the presence of Ag at an energy of 2.98 eV despite the quantitative evaluation displaying a value of zero.

The SEM image of S5 is shown in Fig. 1(E), whose inset is a low-magnification SEM image of S5. With increased AgNO_3_ amount in dipping solution and dipping times, the morphology of the SnO_2_ NWs slightly changed. More tiny particles can be seen in the SEM image of S5, but the sample maintained its entangled NW morphology. Whether the Ag_2_O nanoparticles continuously or discontinuously decorated the surface of SnO_2_ NWs was difficult to observe simply by SEM observation. However, the surface of the samples was found to have increased roughness with increased Ag_2_O decoration. EDS composition analysis of S5 [Fig. 1(F)] showed that the content of Ag was very high (about 3.5 wt%). This result demonstrated that increasing the concentration of AgNO_3_ solution and the dipping times can increase the content of Ag_2_O nanoparticles decorated on the surface of SnO_2_ NWs for effective H_2_S detection.

**Fig. 2:**
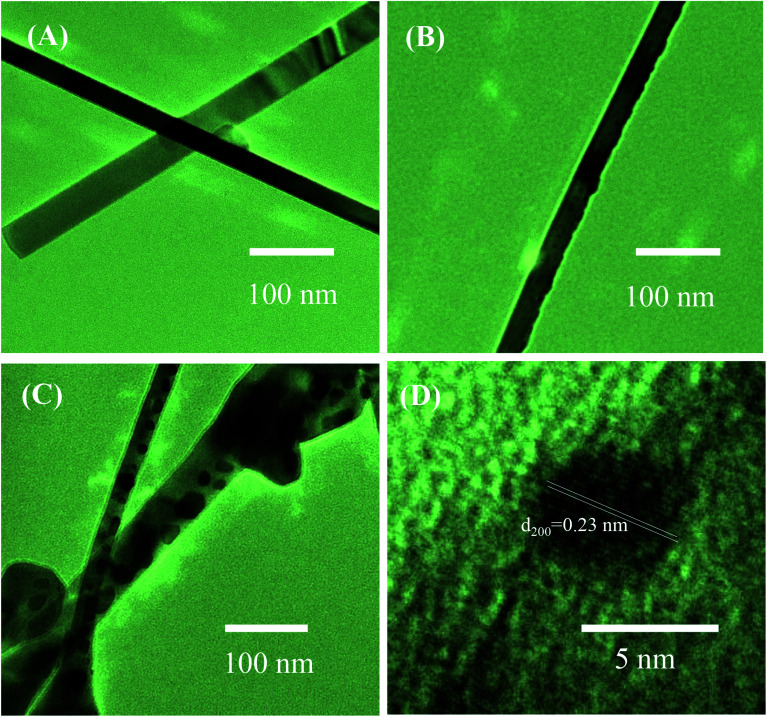
TEM images of SnO_2_ nanowires and SnO_2_ nanowires decorated with Ag_2_O nanoparticles: (A) sensor S1; (B) sensor S2, (C) sensor S5; (D) HRTEM image of Ag_2_O nanoparticle on the surface of nanowire.

To further study the decoration of Ag_2_O on the surface of SnO_2_ NWs, we selected S1, S2, and S5 for TEM characterizations. The grown SnO_2_ NWs had a very smooth and clean surface [Fig. 2(A)]. The average diameter of a SnO_2_ NW was approximately 70 nm, consistently with the observation by SEM images. No Ag_2_O nanoparticle was observed in this sample possibly because the AgNO_3_ concentration of the dipping solution was too low. The HRTEM images of S2 and S5 are shown in Fig. 2(B), and (C), respectively. The black dots decorated on the SnO_2_ NWs surface were Ag_2_O nanoparticles. Given that S2 was decorated by a low concentration of AgNO_3_ (0.2 mM) solution, the density of Ag_2_O nanoparticles on the surface of SnO_2_ NWs was very low [Fig. 2(B)] and the particle sizes were about 7 nm. The size and density of Ag_2_O increased with increased AgNO_3_ concentration (1 mM, 20 times of dipping), as observed in S5 [Fig. 2(C)]. The diameter of Ag_2_O nanoparticles decorated on the surface of SnO_2_ NWs ranged within 5–20 nm. However, they were still smaller than the diameter of SnO_2_ NWs. The wettability of AgNO_3_ solution on the SnO_2_ NW surface is very important for the decoration of Ag_2_O nanoparticles performed by dipping method. Wettability ensures the homogenous decoration of Ag_2_O nanoparticles on the total surface of SnO_2_ NWs. At a high density, Ag_2_O particles may agglomerate and form a large cluster. A high-magnification HRTEM image of about 5 nm Ag_2_O nanoparticle is shown in Fig. 2(D). The interspacing of ∼0.23 nm, which corresponded to the (200) lattice plane of cubic structured Ag_2_O,^42^ was observed. This result was consistent with a previous one on the thermal decomposition of AgNO_3_ at 250–440 °C (ref. 43) into Ag. Then, Ag was oxidized into Ag_2_O at an oxidation temperature of about 350–500 °C.^44^ In the process of e-beam decoration, Ag nanoparticles are anisotropically decorated on one side of NWs but not homogenously.^31^ Herein, the wet chemical method was used to ensure that nanoparticles were homogenously decorated on the surface of the NWs. Notably, S5 had larger Ag_2_O nanoparticles than S2, but decoration was not continuous because overdecoration of Ag_2_O nanoparticles can reduce sensor response.^45^

### 3.2. Gas-sensing characteristics

**Fig. 3:**
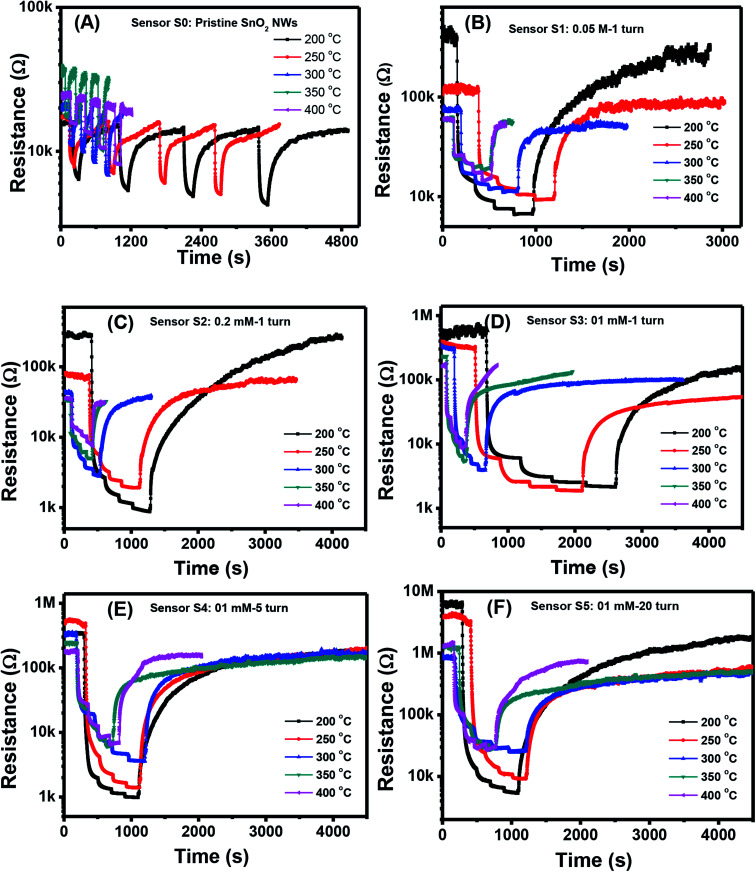
Transient response to different H_2_S concentrations various working temperature of the sample: S0 (A), S1 (B), S2 (C), S3 (D), S4 (E), S5 (F).

Fig. 3(A)–(F) show the changes in transient resistance with time of S0–S5, respectively, upon exposure to various H_2_S concentrations (0.1, 0.25, 0.5, and 1 ppm) measured at different working temperatures (200, 250, 300, 350, and 400 °C). S0 showed significant response to H_2_S at all measured temperatures, but the response and recovery times were very long at low working temperature [Fig. 3(A)]. At a working temperature of 200 °C, S0 required almost 1.5 h to finish one measurement at four concentrations of H_2_S. Thus, stair-type tests were conducted for H_2_S gas sensing because of the slow recovery characteristics [Fig. 3(B)–(F)]. This finding indicated that measurements were conducted through a stepwise increase in H_2_S concentration from 0.1 ppm to 1 ppm before finally being refreshed by dry air. The obtained plots illustrated that the resistance of pristine and decorated SnO_2_ NW sensors steeply increased when H_2_S gas was injected into the test chamber [Fig. 3(B)–(F)]. The resistance then recovered to the initial values when H_2_S was replaced by dry air. All these sensors presented the typical n-type gas-sensing behavior of SnO_2_ NW semiconductor, where resistance decreased with increased H_2_S gas exposure. The base resistance in air of pristine SnO_2_ NWs (S0) was much smaller than that of Ag_2_O-decorated SnO_2_ sensors from S1 to S5. S5, with the largest amount of Ag_2_O decoration, had the highest resistance values in air of about 7 MΩ at 200 °C. Notably, Ag_2_O is also a good conductor, so the high base resistance value of S5 confirmed that the nanoparticles decorated on the surface of SnO_2_ NW formed the p–n heterojunction. Based on the plot of transient resistance *versus* time of the sensors, we roughly estimated that the response values increased but the recovery rate of the sensors decreased with increased Ag_2_O decoration.

**Fig. 4:**
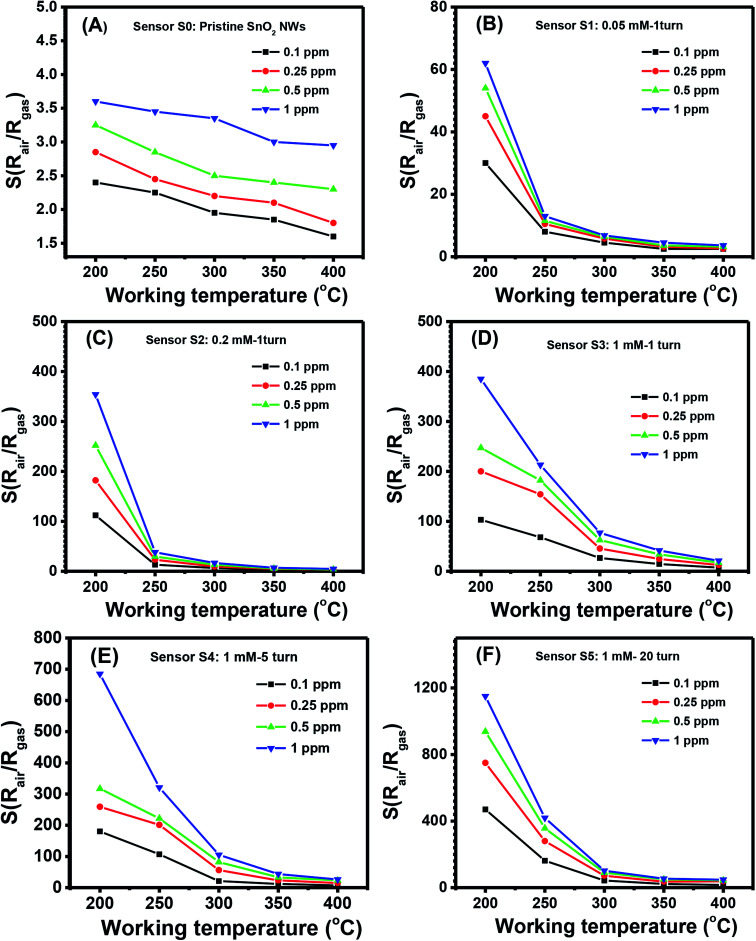
Response to different H_2_S concentrations various working temperature of the sample: S0 (A), S1 (B), S2 (C), S3 (D), S4 (E), S5 (F).

The quantitative response values of different sensors are shown in Fig. 4(A)–(F). The response values of all sensors decreased with increased working temperature within the measured range. This result was similar to that of other metal-oxide-based H_2_S gas sensors.^46^ The pristine SnO_2_ NW sensor (S0) had the highest response value of less than 4 over all the range of working temperatures and gas concentrations [Fig. 4(A)]. The response values for 1 ppm H_2_S decreased almost linearly from 3.6 to 2.9 with increased working temperature from 200 °C to 400 °C. These values were very low compared with the response of Ag_2_O-decorated SnO_2_ NW sensors [Fig. 4(B)–(F)]. The responses of Ag_2_O-decorated SnO_2_ NW sensors from S1 to S5 were much higher than that of pristine SnO_2_ NWs, S0. The responses of Ag_2_O-decorated SnO_2_ NW sensors increased with increased amount of decorated Ag_2_O nanoparticles. All sensors showed better response at lower operating temperatures, reaching the highest values at 200 °C within the measured range. The response values to 1 ppm H_2_S at 200 °C of S1–S5 were approximately 61, 358, 392, 690 and 1155, respectively. The response to 1 ppm H_2_S at 200 °C of S5 was about 320-fold higher than that of S0 under the same measurement condition. Notably, the maximum response to 1 ppm H_2_S gas of rGO-loaded Fe_2_O_3_ nanofibers is only 9.2.^46^ Herein, all sensors had decreased response values with increased working temperature from 200 °C to 400 °C. The response to 0.1 ppm H_2_S at 400 °C of S1 was 2.5, whereas that of S5 was much higher at about 16. Clearly, within the studied H_2_S concentration range (0.1–1 ppm), S5 had the best sensing performance because of its high sensitivity. This finding can be attributed to the p–n heterojunctions between Ag_2_O nanoparticles on the surface and SnO_2_ NWs,^31,47^ similar to the p–n heterojunctions between CuO and SnO_2_ (ref. 19) or NiO and SnO_2_.^21^ Details about the gas-sensing mechanism are discussed in subsequent sections.

**Fig. 5:**
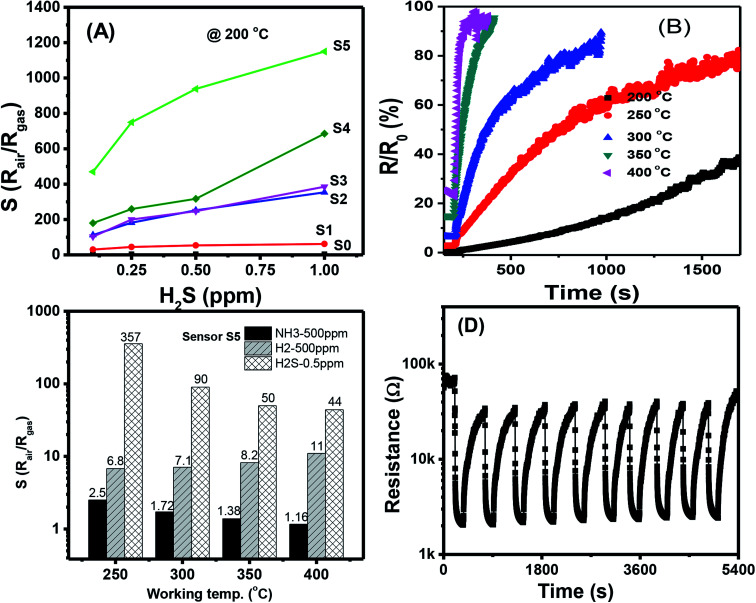
(A) Comparative response of different sensors at 200 °C; (B) response time of sensor S5 measured at different temperatures; (C) response of the sensor S5 at different working temperatures to various gases; (D) stability of the sensor S5 for 10 cycles measured at 250 °C.

The response values of different sensors measured at 200 °C as a function of H_2_S concentration are shown in Fig. 5(A). With a low content of Ag_2_O decoration (S0 to S4), the response values increased almost linearly with H_2_S concentration in the measured range, but their values were low. The highest response value of the sensor S0 at 200 °C for 1 ppm H_2_S is about 3.7. S5 had the highest response values, and the response values increased nonlinearly with H_2_S concentration. Along with the gas response, the recovery time of the sensor is very important in practical applications because it determines sensor reusability. The effects of working temperature on the recovery time of Ag_2_O-decorated SnO_2_ NW sensor are shown in Fig. 5(B). Obviously, the sensor had very poor recovery characteristics at low working temperatures of 200, 250, and 300 °C, *i.e.*, resistance did not recover to the initial value after refreshing for 1000 s. However, the sensor presented 100% recovery characteristics at working temperatures of 350 and 400 °C, with a recovery time of approximately 70 s. In practical application, balance should be achieved between sensor sensitivity and recovery depending on the objective of the application. For instance, the sensors based on 2D materials have poor recovery characteristics, but they could operate at room temperature, thus suitable for low power consumption devices.^48,49^ Herein, the long recovery time is possible due to the formation enthalpy of Ag_2_S (−32.6 kJ mol^−1^) is lower than that of Ag_2_O (−31 kJ mol^−1^), thus it requires higher energy to break the bonding of Ag_2_S than that of Ag_2_O compound. As a result, the sensor has longer recovery time than the response time. The selectivity of S5 toward three reducing gases H_2_S, NH_3_, and H_2_ was tested, and the results are shown in Fig. 5(C). At a low working temperature of 200 °C, the sensor did not show good recovery to H_2_S, so we tested the selectivity at 250, 300, 350, and 400 °C. Results demonstrated that S5 had the highest response toward 0.5 ppm H_2_S despite the 1000-fold concentration in all working temperatures. At a working temperature of 400 °C, S5 still had a high response value of 44–0.5 ppm H_2_S, whereas the corresponding values for 500 ppm NH_3_ and 500 ppm H_2_ were 1.16 and 11, respectively. Reproducibility and repeatability are also important properties of a gas sensor; thus, we tested the short-term stability of the sensor by switching on/off the ambient from air to 0.25 ppm H_2_S gas and back to air at a working temperature of 250 °C. As shown in Fig. 5(D), excepted for the first cycle, the sensor exhibited good recovery characteristics for 10 pulses of measurement, where the base resistance recovered to the initial value after refreshing the chamber with air. The relative standard deviation (RSD) was calculated by the equation 100 × *S*/|*x̄*|, where *S* is the sample standard deviation, *x̄* is sample mean. The RSD value of the sensor for ten pulses measurement is 92.4%, indicating the good reproducibility of the device. However, for real application, long term stability of the sensor should be studied. This work will be characterized in next step, and the data will be reported elsewhere.

For a better vision, the H_2_S sensing performances of the sensors based on functionalized-SnO_2_ nanomaterials are summarized in Table 3. Compared to other results in the references, our sensor showed comparable working temperature whereas was superior in response toward much lower concentration. This means that the Ag_2_O decoration on the surface of SnO_2_ NWs is suitable for development of high performance H_2_S gas sensor.

**Table 3:** A comparative result on the functionalized-SnO_2_ gas sensors for H_2_S detection

Material	Conc. (ppm)	Working temp (°C)	Response (*R*_a_/*R*_g_)	Response/recover*y* times (s)	Ref.
Ag_2_O–SnO_2_ thin film	50	74	99^a^	>600/4500	35
CuO–SnO_2_ NWs	80	300	1280	1/828	23
NiO–SnO_2_ NWs	10	300	1372	11/102	21
CuO–SnO_2_ NWs	10	250	26.3	180/600	19
CuO–SnO_2_ nanofibers	10	150	3000	2/3000	25
CuO–SnO_2_ thin film	100	180	25.3	10/42	22
CuO–SnO_2_ hollow spheres	1	300	22.4	500/1000	50
Ag_2_O–SnO_2_ mesoporous	1	100	71.5	390/1600	33
NiO–SnO_2_ nanoweb	100	300	∼6	N/A	26
NiO–SnO_2_ thin film	10	Room	440	2000/30 000	27
Ag_2_O–SnO_2_ NWs	0.5	—	21	12/1000	51
SnO_2_ NWs	1	—	∼3.5	50/200	14
**Ag**_**2**_ **O–SnO**_**2**_ **NWs**	**1**	**200**	**1150 (S5)**	**350/4000**	**This work**
			**60 (S1)**	**200/1500**	

a *S* = (*R*_air_ − *R*_gas_)/*R*_air_.

### 3.3. Gas-sensing mechanism

The gas-sensing mechanism of a metal oxide-based sensor is determined by the surface reaction of the analyzed gas molecule and pre-adsorbed oxygen species.^9^ When SnO_2_ was exposed to air, atmospheric oxygen molecules were adsorbed on the surface of SnO_2_ NWs to form oxygen ions (O_2_^−^, O^−^, and O^2−^) by withdrawing electrons from the conduction band of SnO_2_, as shown in the following eqn (1)–(3):

1 O_2(gas)_ + e^−^ ↔ O_2_^−^_(ads)_

2 O_2_^−^ + e^−^ ↔ 2O^−^

3 O^−^ + e^−^ ↔ O^2−^

As shown in the above equations, the resistance of SnO_2_ in air increased because of the formation of a thick conduction-depletion region. When air was replaced by H_2_S, the oxygen ions reacted with H_2_S to form SO_2_ and H_2_O and then released electrons back to the conduction band, resulting in decreased SnO_2_ resistance, as presented in eqn (4)–(6):

4 2H_2_S + 3O_2_^−^ ↔ 2SO_2_ + 2H_2_O + 6e^−^

5 H_2_S + 3O^−^ ↔ SO_2_ + H_2_O + 3e^−^

6 H_2_S + 3O^2−^ ↔ SO_2_ + H_2_O + 6e^−^

However, the chemophysical processes of decoration with silver and silver oxide involved in the gas-sensing properties of metal oxides can be explained in various ways.^30,52–54^ The mechanisms are primarily electronic and/or chemical sensitization. The electronic mechanism is related to the extension of the electron-depleted space charge region at the interface between two materials, and the latter is related to the dominance of the dissociation of gas molecules on the surface of decorated materials by spillover effect.^53,54^

**Fig. 6:**
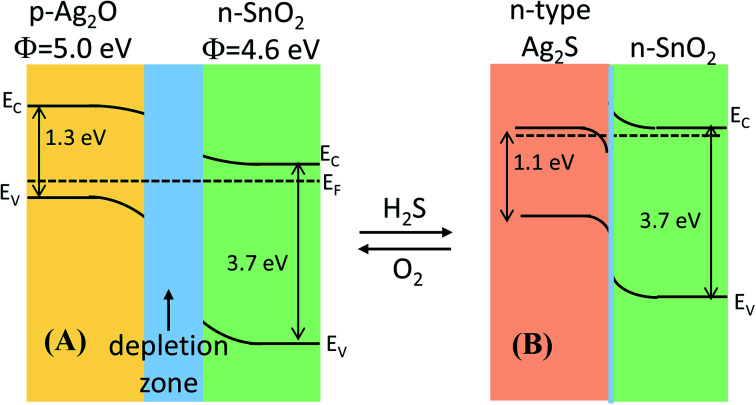
Energy band diagram of the formation of the p-Ag_2_O/n-SnO_2_ junction in air and n-Ag_2_S/n-SnO_2_ in H_2_S atmosphere.

Herein, we believed that the dissociation of gas molecules at Ag-based sites on the surface of Ag-decorated SnO_2_ facilitated the charge-transfer reaction between sensor surface and H_2_S molecule. The gas-sensing mechanism of Ag_2_O-decorated SnO_2_ NWs may involve the variation in band structure caused by the conversion of Ag_2_O into Ag_2_S and back to Ag_2_O when the test ambient switched from air to H_2_S and back to air, as shown in Fig. 6(A) and (B), respectively. Ag_2_O is a p-type narrow band-gap semiconductor (1.3 eV)^47^ with a work function of 5.0 eV,^55,56^ whereas SnO_2_ is a n-type wide direct-band-gap (3.7 eV) semiconductor with a higher work function of 4.6 eV.^57^ Given the extension of the electron-depleted region underneath Ag_2_O nanoparticles on the surface of SnO_2_ NWs, the barrier at the interface between these two materials developed much more than usual.^31^ Furthermore, the formation of a continuous series of n–p–n junctions by decorating Ag_2_O nanoparticles on the network of SnO_2_ NWs, which prevented the electron current in SnO_2_ NWs, aggravated the decrease in SnO_2_ conductivity.^21^ Upon exposure to H_2_S, Ag_2_O was converted into Ag_2_S^58^ according to eqn (7).

7 Ag_2_O + H_2_S ↔ Ag_2_S + H_2_O

The conversion of Ag_2_O into AgS_2_ occurred spontaneously because of the negative free Gibbs energy of the reaction (−224.7 kJ mol^−1^) at room temperature. Therefore, the conversion Ag_2_O into Ag_2_S requires less H_2_S gas, thus the sensor has a lower detection limit. In addition, Ag_2_S can be an n- or p-type semiconductor depending on its surrounding environment and the pressure.^59,60^ The monoclinic α-Ag_2_S is a n-type semiconductor with a band gap of ∼1.1 eV and a work function of 4.42 eV. Upon exposure to H_2_S, the conversion of p-type Ag_2_O^58^ into n-type Ag_2_S destroyed the p–n junctions of Ag_2_O–SnO_2_ and formed the n–n of Ag_2_S–SnO_2_, resulting in largely decreased resistance [Fig. 6(B)]. Ag_2_S was then re-oxidized when the sensor was in air and the p–n junctions were re-established, and the sensor resistance thus recovered to its initial value. Hence, the functionalization of silver on the surface of SnO_2_ NWs improved their H_2_S-sensing properties.

## 4. Conclusion

We introduced a dip-coating method of decorating Ag_2_O nanoparticles on the surface of on-chip-grown SnO_2_ NW sensors toward H_2_S gas monitoring. The effect of Ag_2_O nanoparticles decorated on the surface of SnO_2_ NWs on H_2_S gas-sensing performance was investigated. SnO_2_ NW sensor decorated with Ag_2_O nanoparticles illustrated the highest response of 1150 to 1 ppm H_2_S at a working temperature of 200 °C with reasonable response and recovery time. Selectivity tests over high concentrations of NH_3_ (500 ppm) and H_2_ (500 ppm) at various working temperatures presented excellent response, selectivity, and reproducibility, demonstrating the sensor's potential application in the selective monitoring of low-level H_2_S gas. The high performance of the sensor was also confirmed under the light of sulfurization, which turned the band structure from p–n of Ag_2_O–SnO_2_ into n–n of Ag_2_S–SnO_2_.

## Conflicts of interest

The authors hereby declare that they have no conflict of interests regarding the publication of this paper.

## Supplementary Material
